# Health Literacy and Its Association with the Adoption of the Mediterranean Diet: A Cross-Sectional Study

**DOI:** 10.3390/nu16142176

**Published:** 2024-07-09

**Authors:** Ana Duarte, Juliana Martins, Cristiana Lopes, Maria José Silva, Cláudia Augusto, Silvana Peixoto Martins, Rafaela Rosário

**Affiliations:** 1 Health Sciences Research Unit: Nursing (UICISA: E), Nursing School of Coimbra, Avenida Bissaya Barreto, Polo C, 3046-851 Coimbra, Portugal; anacspduarte@gmail.com (A.D.); julianacmmartins18@gmail.com (J.M.); cristiana.usfg@gmail.com (C.L.); mjsilva@ese.uminho.pt (M.J.S.); coliveira@ese.uminho.pt (C.A.); silvana.martins12@gmail.com (S.P.M.); 2 School of Nursing, University of Minho, Edifício 4, Campus de Gualtar, 4710-057 Braga, Portugal; 3 Research Centre on Nursing (CiEnf), School of Nursing, University of Minho, Edifício 4, Campus de Gualtar, 4710-057 Braga, Portugal; 4 Research Centre on Child Studies (CIEC-UM), Institute of Education, University of Minho, Campus de Gualtar, 4710-057 Braga, Portugal; 5 ProChild CoLAB Against Poverty and Social Exclusion Association, Campus de Couros, Rua Vila Flor, 166, 4810-225 Guimarães, Portugal

**Keywords:** health literacy, health promotion, health care, Mediterranean diet, social vulnerability

## Abstract

Health literacy (HL) is a crucial factor influencing health-related decisions, including dietary patterns. The Mediterranean diet is widely recognized as one of the healthiest eating patterns. This study aims to explore the association between HL levels among adults in socially vulnerable contexts and their adoption of the Mediterranean diet. Conducted as part of a cluster-randomized trial in primary schools, the research involved 557 parents of children aged 6 to 10 who consented to participate. HL was assessed using the HLS questionnaire, while adoption of the Mediterranean diet was evaluated using the MEDAS questionnaire. Participants also provided anthropometric and sociodemographic data via a questionnaire, from which BMI was calculated (weight/height^2^). Education level was categorized as higher education or lower and professional status was classified based on occupational categories. Generalized linear models and logistic regression were employed for analysis. The findings indicate a direct association between HL level and adoption of the Mediterranean diet (B = 0.022, 95% CI 0.010–0.035, *p* < 0.001), underscoring the influence of HL on dietary choices. Specifically, the healthcare dimension of HL emerges as pivotal in shaping dietary behaviors, particularly towards the Mediterranean diet. These results underscore the importance of policies and programs aimed at enhancing HL, especially among socially vulnerable populations. Prioritizing public health interventions to improve HL is critical for promoting better dietary decision-making.

## 1. Introduction

Health literacy (HL) has been widely acknowledged as a pivotal factor in public health [1]. It encompasses the ability to seek, comprehend, and apply health-related information in everyday life [2]. Beyond merely receiving information and scheduling appointments, HL involves empowering individuals to effectively utilize their knowledge [3,4].

In Europe, interest has grown in measuring HL in relation to public health, as monitoring HL can inform professional and political decision-making aimed at improving population health [5]. In 2012, Sørensen and colleagues, as part of the Consortium of the European Health Literacy Project, developed a multidimensional questionnaire, the HLS-EU-Q47, to measure HL. This tool has since been adapted as the HLS19-EU-Q47 by the Action Network on Measuring Population and Organizational Health Literacy (M-POHL) [4,6]. The HLS19-EU-Q47 includes two shortened versions, namely the HLS19-Q12 and HLS19-Q16, with 12 and 16 items, respectively. Each version assesses health literacy across the three domains of health care, health promotion, and disease prevention. Additionally, they evaluate the following four aspects of health-related information management: finding/accessing, understanding, evaluating, and applying health information [2,6].

In Portugal, Arriaga and colleagues have reported high levels of HL [6]. HL has been linked to improved health decision-making and is recognized as a key social determinant of health behaviors [7]. In the context of nutrition, the effective management of information and decision-making processes related to food selection, grocery shopping, meal preparation, and dietary habits can profoundly influence individuals’ health and well-being [7,8].

Dietary choices significantly impact individuals’ health outcomes. Among recognized healthy diets, the Mediterranean diet has garnered considerable attention in recent years [9]. Adoption of the Mediterranean diet has been associated with numerous beneficial health outcomes, benefiting not only individuals [10,11] but also entire families [12]. This dietary pattern is characterized by daily consumption of fruits, olive oil, vegetables, legumes, nuts, and whole grains. Additionally, it includes weekly intake of fish and poultry. It typically involves limited consumption of red meat and moderate alcohol intake, often consumed in moderation with meals [11,12].

The adoption of the Mediterranean diet has been associated with socioeconomic status, as individuals from households with moderate to high socioeconomic status tend to embrace this dietary pattern more frequently [13]. Conversely, individuals from disadvantaged social and economic backgrounds often exhibit lower levels of HL [14]. Factors such as poverty and limited access to fresh foods have been identified as contributors to health inequalities [8].

Some studies have already analyzed the association between HL and the Mediterranean diet [15,16]. However, it is evident that demographic and social characteristics influence both HL and adoption of the Mediterranean diet [15]. To date, little attention has been given to understanding these associations among individuals in vulnerable conditions. Therefore, this study aims to investigate the association between HL levels among adults in socially vulnerable contexts and their adoption of the Mediterranean diet.

## 2. Materials and Methods

### 2.1. Study Design and Ethics

This is an observational cross-sectional analytic study that is part of a cluster-randomized trial (BeE-school Project), which was implemented in primary schools in socially vulnerable contexts. Outcome and exposure were measured at the same time. A total of 557 parents of children aged 6 to 10 participated in the study.

Participants in this cross-sectional study were selected according to the inclusion and exclusion criteria set for the study. The criteria for inclusion encompassed families from primary schools situated in regions marked by economic and social disadvantage, typified by social exclusion and poverty. There were no exclusion criteria concerning parents. A total of ten schools participated in the study. After the participants were selected, we collected data. 

This research project was officially registered in the Clinical Trials database/platform under registration number NCT05395364. Additionally, it obtained ethical approval from the Ethics Subcommittee for Life and Health Sciences (CE.CVS 009/2022) at the University of Minho. The research adhered to the Code of Ethics of the World Medical Association (Declaration of Helsinki). All parents provided informed consent.

### 2.2. Health Literacy

Parents’ HL was evaluated using the short version of the European Health Literacy Survey Questionnaire, with 12 items (HLS_19_-Q12). The HLS_19_-Q12 appears to be a suitable questionnaire for conducting HL screenings [1]. The scale comprises 12 items rated on a Likert-type scale, ranging from 1 for “very difficult” to 4 for “very easy”, with the additional response option of “don’t know” recoded as a missing value during the analysis.

This version has been validated for the Portuguese population [6], enabling assessment of overall HL, including its three domains—healthcare, health promotion, and disease prevention—as well as four aspects related to the management of health-related information. For the present study, general HL and the three specific domains were analyzed.

General HL was calculated as the sum of the items of the core HL measurement items standardized to a 0–100 scale.

The dimensions of HL were calculated with the grouping of items corresponding to health care, health promotion, and disease prevention. Health care includes items 1 to 4, disease prevention includes items 5 to 8, and health promotion includes items 9 to 12 (see Supplementary Table S1 to assess the specific questions for each dimension). The obtained score was also standardized to a 0–100 scale.

Four categorical levels were also identified, namely inadequate (0–25 points or up to 50%), problematic (>25–33 points or 51–66%), sufficient (>33–42 points or 67–80%), and excellent (>42–50 points or 81–100%) [5]. In the present study, the levels of ‘inadequate’ and ‘problematic’ HL were combined.

### 2.3. Adoption of the Mediterranean Diet

Adoption of the Mediterranean diet was assessed using the self-reported Mediterranean Diet Adherence Screener (MEDAS) questionnaire, which is specifically designed to evaluate typical consumption of Mediterranean foods. The questionnaire, comprising fourteen multiple-choice items, has been validated for use in the Portuguese population [11]. To facilitate comprehension with respect to portion sizes, a legend was provided for each question, and any doubts could be clarified by phone or email. 

Responses indicating strong adoption of the Mediterranean diet were scored as one, while those indicating weaker adoption were scored as zero. Scores on the MEDAS questionnaire ranged from zero to fourteen, with a score above nine signifying a high level of adoption of the Mediterranean diet [11]. Using this cut-off, adoption of the Mediterranean diet was categorized as low adoption (scores below or equal to nine) or high adoption (scores exceeding nine). Both the continuous variable (total score) and the categorized variable were used in different statistical analyses. 

### 2.4. Anthropometrics and Sociodemographic Variables

Anthropometric data (weight and height) were self-reported via questionnaire, then converted to the Body Mass Index (BMI), calculated as the ratio of weight to height squared. BMI was utilized as a potential confounding factor in the statistical analyses. For descriptive analysis, BMI was categorized according to WHO recommendations (<18.5, underweight; 18.5–24.9, normal weight; 25.0–29.9, overweight; and >30.0, obesity) [17]. However, due to the reduced number of cases in the underweight category, we combined categories, resulting in three groups, namely normal and underweight, overweight, and obesity.

Sociodemographic variables, including education level and occupation, were recorded via questionnaire. Education level was categorized as either less than secondary educational level, secondary education level, or higher education (university studies). Occupation was initially captured through an open-ended question and subsequently categorized according to the International Standard Classification of Occupations (ISCO), enabling comparison of similar jobs across different countries and vocational training backgrounds [18]. Three occupational categories emerged, namely ‘blue collar’, ‘lower white collar’, and ‘upper white collar’ (see Supplementary Table S2). For statistical analysis, the highest classification within the family was taken into consideration.

### 2.5. Statistical Procedures

Statistical analysis progressed through three distinct steps. Initially, a descriptive analysis was conducted, employing measures of central tendency and dispersion based on the nature of the variables. Categorical variables were presented as frequencies (*n*) and percentages (%), while continuous variables were analyzed descriptively using mean ± standard deviation (SD).

Second, generalized linear models (GLMs) were applied to investigate the general association between general HL and each dimension of HL (predictor) and adoption of the Mediterranean diet (outcome).

Thirdly, a binary logistic regression was conducted to assess the general associations between the adoption of the Mediterranean diet (lower and higher adoption) as an outcome and general HL and each dimension of HL as predictors. The categories of HL (inadequate/problematic, sufficient, and excellent) were further included as predictors, and the adoption of the Mediterranean diet remained an outcome.

In all the analyses (GLM and binary logistic regression), BMI and sociodemographic variables (i.e., education level and occupational classification) were included as covariates. The significance level was set at <0.05. Data analysis was conducted using version 29.0.1 of IBM SPSS^®^.

## 3. Results

### 3.1. Characteristics of Participants

The characterization of the participants is presented in Table 1. The majority of respondents were female (83.7%), with a mean ± SD age of 40.0 ± 5.3 years. Almost half of the participants had a higher education level (48.5%), and 51.4% were classified as upper white-collar workers, the highest occupational status category. Regarding weight status, the mean ± SD BMI of participants was 25.5 ± 4.4. Almost half (53.1%) were normal or underweight, while 46.9% were classified as having overweight or obesity (31.9% and 15.0%, respectively).

The mean ± SD score for HL was 74.1 ± 15.1, with the health promotion dimension having the highest mean ± SD score of 81.8 ± 12.3 points. When HL was categorized into groups, we found that 43.1% of participants had an inadequate/problematic level of literacy. Regarding the adoption of the Mediterranean diet, the mean ± SD score was 7.7 ± 2.1, with 79.7% of participants showing low adoption of this diet.

### 3.2. Generalized Linear Models Results

Table 2 displays the associations between HL and each dimension of HL (namely, health care, disease prevention, and health promotion) and adoption of the Mediterranean diet. Two adjustment models were employed for our analysis to investigate the relationship between the adoption of the Mediterranean diet and HL. Model 1 was the non-adjusted model, while Model 2 was adjusted for participants’ BMI, level of education, and occupational status.

Higher health literacy was directly linked to the adoption of the Mediterranean diet (B = 0.022, 95% CI 0.010–0.035, *p* < 0.001). Similar results were found between each HL dimension and adoption of the Mediterranean diet. Even when adjusted for potential confounders, the associations remained significant for HL and for all HL dimensions. 

### 3.3. Logistic Binary Results

The associations between HL and categorized adoption of the Mediterranean diet (low and high adoption) are presented in Table 3. The analysis was performed using the same adjustment models used in the GLM analysis. We concluded that individuals with higher HL scores had significantly higher odds of having a higher level of adoption of the Mediterranean diet (odds ratio [OR] 1.020, 95% CI 1.004–1.036, *p* = 0.016). However, this association was not significant when adjusted for confounders. Regarding HL dimensions, only the healthcare dimension remained significantly associated with adoption of the Mediterranean diet, even in the adjusted model (OR 1.026, 95% CI 1.007–1.046, *p* = 0.007, and OR 1.024, 95% CI 1.004–1.044, *p* = 0.019, respectively). When examining HL levels, we found that individuals with sufficient levels of HL had significantly higher odds of having a higher level of adoption of the Mediterranean diet, even in the adjusted model (OR 3.309, 95% CI 1.818–6.022, *p* < 0.001, and OR 3.133, 95% CI 1.703–5.767, *p* < 0.001, respectively). Individuals with an excellent level of HL appeared to have similar results, but only in the non-adjusted model.

## 4. Discussion

The present study found that individuals in socially vulnerable contexts who have higher levels of HL tend to adhere more to the Mediterranean diet. These results are perfectly aligned with other studies, which indicate that higher HL is associated with better food choices [7,8,19]. 

The Mediterranean diet is recognized as one of the healthiest diets and has been associated with improved health outcomes, including a lower incidence of several chronic non-communicable diseases and a weight-loss effect [19,20]. This diet features the consumption of a variety of fresh vegetables and fruits, olive oil as the primary source of dietary fat, regular intake of nuts and seeds, legumes several times per week, daily consumption of whole grains, and fish and seafood two to three times per week. Herbs and spices are used to season meals, while sweets and red/processed meats are consumed less frequently. Water is the main beverage, with wine consumed in small portions during meals by individuals who are accustomed to it and do not have health conditions or take medications that contraindicate alcohol consumption [19]. Despite this, only 20.3% of participants in our study exhibited high levels of adoption of the Mediterranean diet. This low adoption could be associated with various factors, such as low socioeconomic status. According to Moustakim and colleagues [13], individuals from medium to high socioeconomic backgrounds typically exhibit a higher propensity to adopt the Mediterranean diet, contrasting with those from low socioeconomic status households. Furthermore, robust adoption of the Mediterranean diet has been correlated with elevated dietary expenditures [21,22]. Considering the prevalent social vulnerabilities among our sample, this could reasonably account for the observed low adoption of the Mediterranean diet.

On the other hand, health literacy encompasses not only the ability to access but also to comprehend and apply health information to make decisions in everyday life, such as with respect to dietary choices [4,14]. Specifically in nutrition, food literacy appears to play an important role in informed decision-making about what individuals eat and how it affects their health [8,23]. Food literacy encompasses more than just nutritional literacy. It includes not only an individual’s ability to access, process, and understand nutritional information and its impact on health but also other aspects such as culture, environment, food identity, and eating behavior [16,24].

In the present study, we did not specifically evaluate food literacy, but it is interesting that HL, in general, could be, to some extent, associated to what individuals eat. This aligns with existing evidence that identified higher levels of HL with the adoption of healthy lifestyles in general [25,26]. For example, Fauci and collaborators [26] studied the Mediterranean population, specifically inhabitants of Sicily and Calabria, to evaluate their levels of HL. In their observational study, they concluded that lower levels of HL are associated with a poorer understanding of the importance of various lifestyle habits, such as diet, smoking, alcohol consumption, and physical activity [26].

Another finding concerning HL is that nearly 56.9% of participants have sufficient or excellent levels of HL. This could be related to our predominantly female sample (83.7% women), who typically have higher HL levels [27]. According to Chakraverty and collaborators, a study on migraines found that men may have lower health literacy than women. Nevertheless, 43.1% of participants presented an inadequate or problematic HL level, which is above the national average for Portugal, where only 29.5% (22.0% inadequate and 7.5% problematic) of individuals have these HL levels [6].

Another finding from the analysis is the apparent importance of the healthcare dimension of HL in adoption of the Mediterranean diet. In both analyses, this specific dimension remained significantly associated with higher adoption of the Mediterranean diet. This specific dimension refers to “the capability to acquire medical or clinical information, comprehend it, analyze, and assess its relevance, and subsequently make informed decisions regarding medical matters while adhering to medical advice” [2]. Our findings highlight the significant responsibility of healthcare professionals and services in promoting healthy lifestyles, particularly in the area of diet. They also underscore the importance of exploring public health interventions to enhance individuals’ HL. Given that HL is a complex issue with numerous contributing factors [28], it is crucial to consider the contexts and backgrounds that could influence it, especially within the healthcare dimension.

We chose to include education level and occupational status as covariates, as these are important measures that characterize socioeconomic status and are considered key determinants of HL [14,28]. According to Stormacq and colleagues [14], disadvantaged social and socioeconomic conditions contribute to low HL levels, serving as an important mediator in the association between socioeconomic status and health status. Our results align with this finding, showing that 43% of individuals have inadequate or problematic HL levels, consistent with national data for Portugal, which indicate that nearly 49% of adults have inadequate or problematic HL levels [6].

We also included BMI as a measure of adiposity and a potential confounder, despite its association with the Mediterranean diet [19]. While we acknowledge that other measures of adiposity, such as the waist-to-height ratio, could be used, we do not have information on the waist circumference of participants. Additionally, BMI is significantly associated with HL, with the odds of overweight/obesity being higher among individuals with lower levels of HL [29].

Upon analyzing the participants’ BMI, it was discovered that nearly half (46.9%) of them were classified as having overweight or obesity. These results are consistent with national data for Portuguese adults aged 18 to 64, which indicate that 36.5% have overweight and 21.6% have obesity [30]. Additionally, social determinants seem to play an important role in weight status, with individuals from lower socioeconomic backgrounds having a higher probability of having overweight and obesity [31,32].

Furthermore, this sample is predominantly composed of women (83.7%). This is due to the fact that this study is part of a broader research project focusing on children, and in Portugal, mothers are typically the primary caregivers [33]. However, especially among women, there is a tendency to provide more accurate information concerning anthropometric data [34,35]. Therefore, having mostly mothers respond adds greater reliability to the reported data.

This study has several strengths that should be emphasized. Initially, it was integrated into a larger research endeavor, thereby increasing the credibility of our results. Additionally, we performed two different types of analysis to better understand the association between variables, accounting for significant potential confounders such as education level, occupational status, and respondents’ BMI. Moreover, this study adds to the investigation of the connections between health literacy and dietary habits among individuals facing social vulnerability.

Additionally, this study has some limitations. This study has a constrained sample size, potentially limiting the applicability of the results to broader populations. Moreover, all collected data were self-reported, making them subject to bias, including desirable bias. Therefore, the accuracy of the data should be approached with caution. Nonetheless, self-reported data have been widely used in epidemiological studies, and the reliability of the results is still considered possible [6,36,37,38,39]. 

The association of HL among adults in socially vulnerable contexts with their adoption of the Mediterranean diet is significant, particularly when considering the role of the healthcare dimension. Our findings highlight the responsibility of healthcare professionals and emphasize the urgency of implementing public health interventions to enhance HL among the population, especially individuals living in socially vulnerable contexts. 

## 5. Conclusions

This study underscores the need for targeted interventions that improve HL among individuals with vulnerabilities. Enhancing HL can lead to better health behaviors and, consequently, to improved health outcomes by empowering individuals to engage more effectively in their health care, adhere to medical advice, and adopt healthier lifestyles. Moreover, improving HL can help reduce health inequalities by providing disadvantaged groups with the tools they need to manage their health more effectively.

## Figures and Tables

**Table 1:** Participant characteristics.

Characteristics	Total (*n* = 557)
Sex	--
Male	91 (16.3%)
Female	466 (83.7%)
Age (years)	40.0 ± 5.3
Education level	--
Less than secondary level	82 (13.4%)
Secondary educational level	224 (36.7%)
University studies	304 (49.8%)
Occupational status (collar)	--
Blue collar	84 (15.6%)
Lower white collar	178 (33.0%)
Upper white collar	277 (51.4%)
BMI	25.5 ± 4.4
BMI categories	--
Normal and underweight	290 (53.1%)
Overweight	174 (31.9%)
Obesity	82 (15.0%)
HL (total standardized to 0–100)	74.1 ± 15.1
Health promotion	81.8 ± 12.3
Disease prevention	78.6 ± 13.0
Health care	80.2 ± 12.9
HL groups	--
Inadequate/problematic	197 (43.1%)
Sufficient	135 (29.5%)
Excelent	125 (27.4%)
MEDAS	7.7 ± 2.1
Low adoption	388 (79.7%)
High adoption	99 (20.3%)

Values are expressed as mean ± SD for continuous variables and *n* (%) for categorical variables.

**Table 2:** Associations between HL and adoption of the Mediterranean diet (GLM analysis).

	MEDAS Score	
	Model 1	Model 2
HL total score	0.02 (0.01; 0.04) *	0.02 (0.01; 0.03) *
Health care	0.03 (0.02; 0.05) *	0.03 (0.01; 0.04) *
Disease prevention	0.02 (0.01; 0.04) *	0.02 (0.004; 0.03) *
Health promotion	0.02 (0.01; 0.04) *	0.02 (0.004; 0.04) *

Note: Values expressed as B (CI 95%). Model 1—non adjusted model; Model 2—model adjusted for respondent BMI, education level, and occupational status. * *p*-value < 0.05.

**Table 3:** Association between HL and adoption of the Mediterranean Diet (binary logistic regression).

	MEDAS Adoption	
	Model 1	Model 2
HL total score	1.02 (1.00; 1.04) *	1.02 (1.00; 1.03)
Health care	1.03 (1.01; 1.05) *	1.02 (1.00; 1.04) *
Disease prevention	1.02 (1.00; 1.04)	1.01 (0.99; 1.03)
Health promotion	1.02 (1.00; 1.04) *	1.02 (1.00; 1.04)
HL levels		
Inadequate/problematic	Ref.	Ref.
Sufficient	3.31 (1.82; 6.02) *	3.13 (1.70; 5.77) *
Excelent	1.95 (1.03; 3.68) *	1.57 (0.82; 3.03)

Note: Values expressed as odds ratios (ORs) (CI 95%). Model 1—non adjusted model; Model 2—model adjusted for respondent BMI, education level, and occupational status. * *p*-value < 0.05.

## Data Availability

The original contributions featured in this study are accessible within the article/Supplementary Materials. Additional queries may be directed to the corresponding author.

## Supplementary Materials

The following supporting information can be downloaded at: https://www.mdpi.com/article/10.3390/nu16142176/s1, Supplementary Table S1. Questions for each dimension from the European Health Literacy Survey Questionnaire (HLS19-Q12); Supplementary Table S2. Occupational categories according to International Standard Classification of Occupations (ISCO).

## References

[1] Finbråten H.S., Wilde-Larsson B., Nordström G., Pettersen K.S., Trollvik A., Guttersrud Ø. (2018). Establishing the HLS-Q12 Short Version of the European Health Literacy Survey Questionnaire: Latent Trait Analyses Applying Rasch Modelling and Confirmatory Factor Analysis. BMC Health Serv. Res..

[2] Sørensen K., Van den Broucke S., Pelikan J.M., Fullam J., Doyle G., Slonska Z., Kondilis B., Stoffels V., Osborne R.H., Brand H. (2013). Measuring Health Literacy in Populations: Illuminating the Design and Development Process of the European Health Literacy Survey Questionnaire (HLS-EU-Q). BMC Public Health.

[3] Nutbeam D. (1998). Health Promotion Glossary. Health Promot. Int..

[4] Sørensen K., Van den Broucke S., Fullam J., Doyle G., Pelikan J., Slonska Z., Brand H. (2012). Health Literacy and Public Health: A Systematic Review and Integration of Definitions and Models. BMC Public Health.

[5] The HLS19 Consortium of the WHO Action Network M-POHL (2021). International Report on the Methodology, Results, and Recommendations of the European Health Literacy Population Survey 2019–2021 (HLS19) of M-POHL.

[6] Arriaga M., Francisco R., Nogueira P., Oliveira J., Silva C., Câmara G., Sørensen K., Dietscher C., Costa A. (2022). Health Literacy in Portugal: Results of the Health Literacy Population Survey Project 2019–2021. Int. J. Environ. Res. Public Health.

[7] Zwierczyk U., Sowada C., Duplaga M. (2022). Eating Choices—The Roles of Motivation and Health Literacy: A Cross-Sectional Study. Nutrients.

[8] Silva P., Araújo R., Lopes F., Ray S. (2023). Nutrition and Food Literacy: Framing the Challenges to Health Communication. Nutrients.

[9] Cobo-Cuenca A.I., Garrido-Miguel M., Soriano-Cano A., Ferri-Morales A., Martínez-Vizcaíno V., Martín-Espinosa N.M. (2019). Adherence to the Mediterranean Diet and Its Association with Body Composition and Physical Fitness in Spanish University Students. Nutrients.

[10] Guasch-Ferré M., Salas-Salvadó J., Ros E., Estruch R., Corella D., Fitó M., Martínez-González M.A., Arós F., Gómez-Gracia E., Fiol M. (2017). The PREDIMED Trial, Mediterranean Diet and Health Outcomes: How Strong Is the Evidence?. Nutr. Metab. Cardiovasc. Dis..

[11] Gregório M.J., Rodrigues A.M., Salvador C., Dias S.S., de Sousa R.D., Mendes J.M., Coelho P.S., Branco J.C., Lopes C., Martínez-González M.A. (2020). Validation of the Telephone-Administered Version of the Mediterranean Diet Adherence Screener (Medas) Questionnaire. Nutrients.

[12] Duarte A., Martins J., Silva M.J., Augusto C., Martins S.P., Rosário R. (2024). The Role of Parental Adherence to the Mediterranean Diet and Family Time Together in Children’s Weight Status: The BeE-School Project. Nutrients.

[13] Moustakim R., Mziwira M., El-Ayachi M., Belahsen R. (2023). Sociodemographic, Nutritional and Health Status Factors Associated with Adherence to Mediterranean Diet in an Agricultural Moroccan Adult’s Population. Rocz. Panstw. Zakl. Hig..

[14] Stormacq C., Van den Broucke S., Wosinski J. (2019). Does Health Literacy Mediate the Relationship between Socioeconomic Status and Health Disparities? Integrative Review. Health Promot. Int..

[15] Albani E.N., Togas C., Kanelli Z., Fradelos E.C., Mantzouranis G., Saridi M., Tzenalis A. (2022). Is There an Association between Health Literacy and Adherence to the Mediterranean Diet? A Cross-Sectional Study in Greece. Wiad. Lek..

[16] Abreu F., Hernando A., Goulão L.F., Pinto A.M., Branco A., Cerqueira A., Galvão C., Guedes F.B., Bronze M.R., Viegas W. (2023). Mediterranean Diet Adherence and Nutritional Literacy: An Observational Cross-Sectional Study of the Reality of University Students in a COVID-19 Pandemic Context. BMJ Nutr. Prev. Health.

[17] Expert Panel on the Identification, Evaluation, and Treatment of Overweight in Adults (1998). Clinical Guidelines on the Identification, Evaluation, and Treatment of Overweight and Obesity in Adults: Executive Summary. Am. J. Clin. Nutr..

[18] Pförtner T.-K., Günther S., Levin K.A., Torsheim T., Richter M. (2015). The Use of Parental Occupation in Adolescent Health Surveys. An Application of ISCO-Based Measures of Occupational Status. J. Epidemiol. Community Health.

[19] Dominguez L.J., Veronese N., Di Bella G., Cusumano C., Parisi A., Tagliaferri F., Ciriminna S., Barbagallo M. (2023). Mediterranean Diet in the Management and Prevention of Obesity. Exp. Gerontol..

[20] Dominguez L.J., Di Bella G., Veronese N., Barbagallo M. (2021). Impact of Mediterranean Diet on Chronic Non-Communicable Diseases and Longevity. Nutrients.

[21] Mendonça N., Gregório M.J., Salvador C., Henriques A.R., Canhão H., Rodrigues A.M. (2022). Low Adherence to the Mediterranean Diet Is Associated with Poor Socioeconomic Status and Younger Age: A Cross-Sectional Analysis of the EpiDoC Cohort. Nutrients.

[22] Tong T.Y.N., Imamura F., Monsivais P., Brage S., Griffin S.J., Wareham N.J., Forouhi N.G. (2018). Dietary Cost Associated with Adherence to the Mediterranean Diet, and Its Variation by Socio-Economic Factors in the UK Fenland Study. Br. J. Nutr..

[23] Silva P. (2023). Food and Nutrition Literacy: Exploring the Divide between Research and Practice. Foods.

[24] Torres R., Real H. (2021). Literacia Nutricional e Literacia Alimentar: Uma Revisão Narrativa Sobre Definição, Domínios e Ferramentas de Avaliação. Acta Port. Nutr..

[25] Wieczorek M., Meier C., Kliegel M., Maurer J. (2023). Relationship Between Health Literacy and Unhealthy Lifestyle Behaviours in Older Adults Living in Switzerland: Does Social Connectedness Matter?. Int. J. Public Health.

[26] Fauci V., Trimarchi G., Ceccio C., Mazzitelli F., Pappalardo R., Alessi V. (2022). Health Literacy in Mediterranean General Population. J. Prev. Med. Hyg..

[27] Chakraverty D., Baumeister A., Aldin A., Seven Ü.S., Monsef I., Skoetz N., Woopen C., Kalbe E. (2022). Gender Differences of Health Literacy in Persons with a Migration Background: A Systematic Review and Meta-Analysis. BMJ Open.

[28] Van Hoa H., Giang H.T., Vu P.T., Van Tuyen D., Khue P.M. (2020). Factors Associated with Health Literacy among the Elderly People in Vietnam. Biomed. Res. Int..

[29] Toçi E., Burazeri G., Kamberi H., Toçi D., Roshi E., Jerliu N., Bregu A., Brand H. (2021). Health Literacy and Body Mass Index: A Population-Based Study in a South-Eastern European Country. J. Public Health.

[30] Lopes C., Torres D., Oliveira A., Severo M., Alarcão V., Guiomar S., Mota J., Teixeira P., Rodrigues S., Lobato L. (2017). Inquérito Alimentar Nacional e de Atividade Física, IAN-AF 2015–2016: Relatório de Resultados.

[31] Ortiz-Moncada R., Álvarez-Dardet C., Miralles-Bueno J.J., Ruíz-Cantero M.T., Dal Re-Saavedra M.A., Villar-Villalba C., Pérez-Farinós N., Serra-Majem L. (2011). Determinantes Sociales de Sobrepeso y Obesidad En España 2006. Med. Clin..

[32] Javed Z., Valero-Elizondo J., Maqsood M.H., Mahajan S., Taha M.B., Patel K.V., Sharma G., Hagan K., Blaha M.J., Blankstein R. (2022). Social Determinants of Health and Obesity: Findings from a National Study of US Adults. Obesity.

[33] Martins M., Sarmento T. (2013). Associações de Pais e Participação Coletiva: Oportunidade Perdida?. Gest. Desenvolv..

[34] Lin C.J., DeRoo L.A., Jacobs S.R., Sandler D.P. (2012). Accuracy and Reliability of Self-Reported Weight and Height in the Sister Study. Public Health Nutr..

[35] Lois K., Kumar S., Williams N., Birrell L. (2011). Can Self-Reported Height and Weight Be Relied Upon?. Occup. Med..

[36] Fonseca H., Silva A., Matos M., Esteves I., Costa P., Guerra A., Gomes-Pedro J. (2010). Validity of BMI Based on Self-reported Weight and Height in Adolescents. Acta Paediatr..

[37] Thomas III J., Paulet M., Rajpura J.R. (2016). Consistency between Self-Reported and Recorded Values for Clinical Measures. Cardiol. Res. Pract..

[38] Chia Y.C., Ching S.M., Ooi P.B., Beh H.C., Chew M.T., Chung F.F.L., Kumar N., Lim H.M. (2023). Measurement Accuracy and Reliability of Self-Reported versus Measured Weight and Height among Adults in Malaysia: Findings from a Nationwide Blood Pressure Screening Programme. PLoS ONE.

[39] Maukonen M., Männistö S., Tolonen H. (2018). A Comparison of Measured versus Self-Reported Anthropometrics for Assessing Obesity in Adults: A Literature Review. Scand. J. Public Health.

