# Supplementary material for: Health Literacy and Its Association with the Adoption of the Mediterranean Diet: A Cross-Sectional Study
Source: Nutrients. 2024 Jul 9;16(14):2176. doi: 10.3390/nu16142176 (PMC11280044; doi:10.3390/nu16142176)
Supplement: Supplementary file 1 [file nutrients-16-02176-s001.zip › nutrients-3050103-supplementary.pdf]

Supplementary Table S1

Supplementary Table S1. Questions for each dimension from the European Health Literacy Survey Questionnaire (HLS19-Q12)

**Dimension**	**Item wording**<br>"On a scale from very easy to very difficult, how easy would you say it is…"
**Healthcare**	...to find out where to get professional help when you are ill?<br>[Instructions: such as doctor, nurse, pharmacist, psychologist]<br>...to understand information about what to do in a medical emergency?<br>...to judge the advantages and disadvantages of different treatment options?<br>...to act on advice from your doctor or pharmacist?
**Disease prevention**	...to find information on how to handle mental health problems?<br>[Instruction: stress, depression or anxiety]<br>...to understand information about recommended health screenings or examinations?<br>[Instructions: e.g., colorectal cancer screening, blood sugar test]<br>...to judge if information on unhealthy habits, such as smoking, low physical activity or drinking too much alcohol, are reliable?<br>...to decide how you can protect yourself from illness using information from the mass media?<br>[Instructions: e.g., Newspapers, TV or Internet]
**Health promotion**	...to find information on healthy lifestyles such as physical exercise, healthy food or nutrition?<br>...to understand advice concerning your health from family or friends?<br>...to judge how your housing conditions may affect your health and well-being?<br>...to make decisions to improve your health and well-being?

Supplementary Table S2

ISCO	Categories in this study
**Managers**	Upper White collar
**Professionals**	Lower White collar
**Technicians and Associate Professionals**	Lower White collar
**Clerical Support Workers**	Upper White collar
**Services and Sales Workers**	Lower White collar
**Skilled, Agricultural, Forestry and Fishery Workers**	Blue collar
**Craft and Related Trades Workers**	Blue collar
**Plant and Machine Operators and Assemblers**	Blue collar
**Elementary Occupations**	Blue collar
**Armed Forces Occupations***	--

*__Note:__ in this study we don't have any case of this occupation category.

Table S3. Associations between HL and adherence to the Mediterranean diet (GLM analysis).

MEDAS score			
	**Model 1**	**Model 2**	**Modelo 3**
**HL total score**	**0.02 (0.01; 0.04)***	**0.02 (0.01; 0.03)***	**0.02 (0.01; 0.03)***
**Healthcare**	**0.03 (0.02; 0.05)***	**0.03 (0.01; 0.04)***	**0.03 (0.01; 0.04))***
**Disease prevention**	**0.02 (0.01; 0.04)***	**0.02 (0.004; 0.03)***	**0.02 (0.003; 0.03)***
**Health promotion**	**0.02 (0.01; 0.04)***	**0.02 (0.004; 0.04)***	**0.02 (0.001; 0.03)***

Note: Values expressed as B (CI 95%). ***p-value** <0.05. **Model 1** – non adjusted model; **Model 2** – adjusted model for respondent's BMI, education level and occupational status. **Model 3** – adjusted model for respondent's BMI, education level, occupational status and **family income**.

Table S4. Association between HL and adherence to the Mediterranean diet (binary logistic analysis).

MEDAS adherence			
	**Model 1**	**Model 2**	**Modelo 3**
**HL total score**	**1.02 (1.00; 1.04)***	1.02 (1.00; 1.03)	1.02 (1.00; 1.04)
**Healthcare**	**1.03 (1.01; 1.05)***	**1.02 (1.00; 1.04)***	**1.02 (1.00; 1.05)***
**Disease prevention**	1.02 (1.00; 1.04)	1.01 (0.99; 1.03)	1.01 (0.99; 1.03)
**Health promotion**	**1.02 (1.00; 1.04)***	1.02 (1.00; 1.04)	1.02 (1.00; 1.04)
**HL levels**			
Inadequate/problematic	Ref.	Ref.	Ref.
Sufficient	**3.31 (1.82; 6.02)***	**3.13 (1.70; 5.77)***	**2.93 (1.53; 5.617)***
Excelent	**1.95 (1.03; 3.68)***	1.57 (0.82; 3.03)	1.61 (0.79; 3.30)

Note: Values expressed as odds ratio (OR) (CI 95%). ***p-value** <0.05. **Model 1** – non adjusted model; **Model 2** – adjusted model for respondent's BMI, education level and occupational status. **Model 3** – adjusted model for respondent's BMI, education level, occupational status and **family income**.
